# Proximity extension assay in cerebrospinal fluid identifies neurofilament light chain as biomarker of neurodegeneration in sporadic cerebral amyloid angiopathy

**DOI:** 10.1186/s13195-024-01473-0

**Published:** 2024-05-14

**Authors:** Marc Vervuurt, H. Bea Kuiperij, Anna M. de Kort, Iris Kersten, Catharina J. M. Klijn, Floris H. B. M. Schreuder, Marcel M. Verbeek

**Affiliations:** 1 Donders Institute for Brain, Cognition and Behaviour, Department of Neurology, Radboud University Medical Center, Nijmegen, The Netherlands; 2 Department of Human Genetics, Radboud University Medical Center, P.O. Box 9101, Nijmegen, 830 TML, 6500 HB The Netherlands

**Keywords:** Cerebral amyloid angiopathy, Cerebrospinal fluid, Proteomics, Neurofilament light chain, Proximity extension assay, Biomarkers

## Abstract

**Background:**

Sporadic cerebral amyloid angiopathy (sCAA) is a disease characterised by the progressive deposition of the amyloid beta (Aβ) in the cerebral vasculature, capable of causing a variety of symptoms, from (mild) cognitive impairment, to micro- and major haemorrhagic lesions. Modern diagnosis of sCAA relies on radiological detection of late-stage hallmarks of disease, complicating early diagnosis and potential interventions in disease progression. Our goal in this study was to identify and validate novel biomarkers for sCAA.

**Methods:**

We performed a proximity extension assay (PEA) on cerebrospinal fluid (CSF) samples of sCAA/control participants (*n* = 34/51). Additionally, we attempted to validate the top candidate biomarker in CSF and serum samples (*n* = 38/26) in a largely overlapping validation cohort, through analysis with a targeted immunoassay.

**Results:**

Thirteen proteins were differentially expressed through PEA, with top candidate NFL significantly increased in CSF of sCAA patients (*p* < 0.0001). Validation analyses using immunoassays revealed increased CSF and serum NFL levels in sCAA patients (both *p* < 0.0001) with good discrimination between sCAA and controls (AUC: 0.85; AUC: 0.79 respectively). Additionally, the CSF: serum NFL ratio was significantly elevated in sCAA (*p* = 0.002).

**Discussion:**

Large-scale targeted proteomics screening of CSF of sCAA patients and controls identified thirteen biomarker candidates for sCAA. Orthogonal validation of NFL identified NFL in CSF and serum as biomarker, capable of differentiating between sCAA patients and controls.

**Supplementary Information:**

The online version contains supplementary material available at 10.1186/s13195-024-01473-0.

## Background

Cerebral amyloid angiopathy (CAA) is an age-related, progressive cerebrovascular disorder characterized by the accumulation of amyloid-beta (Aβ) peptides in the walls of cerebral blood vessels [1]. This pathological deposition of Aβ, derived from the proteolytic cleavage of the amyloid precursor protein (APP), disrupts the architecture of the vessel walls. These deposits primarily affect small to medium-sized arteries, arterioles, and capillaries in the brain. The presence of Aβ deposits in the vessel walls renders them more susceptible to rupture, which may result in spontaneous intracerebral haemorrhage as well as other haemorrhagic manifestations [2]. In addition to the risk of haemorrhage, CAA has been associated with cognitive impairment and dementia. CAA is known to coincide with other neurodegenerative diseases, including Alzheimer’s disease (AD), which may complicate the clinical picture [3].

The clinical diagnosis of CAA can be challenging due to its overlapping features with other (neurodegenerative) diseases, such as AD and deep perforating vasculopathy. Definitive diagnosis can only be made based on pathological analysis of brain tissue, which complicates sCAA diagnosis in life. Neuroimaging techniques (including magnetic resonance imaging (MRI) and computed tomography) are able to diagnose sCAA with relatively high probability by visualizing characteristic features associated with sCAA, such as lobar cerebral microbleeds, cortical superficial siderosis, and convexity subarachnoid haemorrhages [4]. However, these imaging modalities are only capable of diagnosing sCAA in a late stage of disease, necessitating the development of more specific diagnostic tools to diagnose disease in earlier stages of disease [5, 6]. To address this challenge, biomarkers for CAA may be found in cerebrospinal fluid (CSF) [7]. Untargeted and unbiased approaches, such as untargeted mass spectrometry-based proteomics and larger multiplex protein arrays, have revolutionized the large-scale screening of protein biomarker candidates for all kinds of (neurodegenerative) diseases [8].

In this study, we have applied a targeted, multiplex proximity extension assay (PEA) to CSF of sporadic cerebral amyloid angiopathy (sCAA) patients and control subjects. PEA enables simultaneous measurement of a vast set of protein biomarkers in CSF. Additionally, we have attempted to validate these findings using targeted immunoassays for the most prominent biomarker candidate, neurofilament light chain (NFL).

## Methods

### Human subjects

sCAA patients (*n* = 44) were included at the Radboud University Medical Center (RUMC) in Nijmegen, the Netherlands. Most of the sCAA patients (*n* = 42) were enrolled through cross-sectional cohort studies (Cerebral Amyloid Angiopathy Vascular Imaging and fluid markers of Amyloid deposition (CAVIA), BIOmarkers for cogNitive Impairment due to Cerebral amyloid angiopathy (BIONIC), aimed at identifying new CSF biomarkers for sCAA at the RUMC (Website: www.radboudumc.nl/BCS) [9–13]. The two remaining sCAA patients were identified through routine diagnostic workflow at the hospital. Participants were included after receiving a diagnosis of probable CAA based on the modified Boston Criteria [14]. All sCAA participants included in this study were diagnosed with probable sCAA and subsequently underwent a comprehensive assessment that included clinical and neuropsychological tests (including the Montreal Cognitive Assessment or MoCA), venipunctures and lumbar punctures, and 3.0 Tesla brain MRI. Further details on MRI are described in [13].

Patients were assessed on the following (small vessel) disease markers: presence of ICH, number and distribution of cerebral microbleeds (CMBs), presence and extent of cortical superficial siderosis (CSS; 0 = no CSS, 1 = focal CSS, 2 = disseminated CSS), presence and extent of enlarged perivascular spaces (EPVS) in the centrum semi-ovale (CSO; using a dichotomized classification: high (≥ 21 EPVS) or low (≤ 20 EPVS)) and white matter hyperintensities (WMH) according to the Fazekas Scale (ranging from 0 to 3). Using these four parameters, we calculated a summary score of SVD markers in sCAA, referred to as CAA-related SVD burden score, ranging from 0 to 6 points [29].

We included 52 control participants in this study. Among them, 27 were enrolled through the CAFE study and underwent exactly the same investigations as the sCAA patients in these studies [13]. Inclusion criteria were age ≥ 55 years, a MoCA score > 28 or a modified Telephone Interview of Cognitive Status (mTICS) score of ≥ 35. Additional exclusion criteria for the controls included self-reported cognitive decline, and a history of major brain pathology such as spontaneous parenchymal intracerebral haemorrhage, ischemic stroke, neurodegenerative disease, brain tumours, brain infection or inflammation. The remaining 25 controls underwent lumbar punctures as part of diagnostic workup of suspected neurologic symptoms or to rule out central nervous system involvement in systemic diseases. None of these 25 participants suffered from the suspected neurological disease, known cognitive impairment, recent stroke (within the last 6 months), sepsis, or central nervous system malignancies.

CSF was collected through means of a lumbar puncture. CSF was collected in polypropylene tubes, centrifuged, aliquoted, and stored in polypropylene tubes at -80 °C. Serum was collected through venipuncture, and collected in polypropylene tubes, centrifuged, aliquoted and stored at -80 °C. This study was performed in accordance with the 1964 Declaration of Helsinki and later amendments and was approved by the Medical Ethics Committee Arnhem-Nijmegen (2014 − 1401, 2017–3810 and 2017–3605 respectively).

### Subject selection for PEA analysis

For the PEA analysis, 34 sCAA patients (*n* = 28 BIONIC, *n* = 4 CAVIA, *n* = 2 through routine diagnostics) and 50 controls (*n* = 25 CAFE, *n* = 25 through routine diagnostics) were analysed. Subjects were age- and sex matched (*p* = 0.84 and *p* = 0.79). Subject characteristics of the exploration study are described in Table 1. CSF biomarkers showed a typical sCAA profile, with decreased levels of Aβ40 and Aβ42 levels in sCAA (both *p* < 0.001), and minor, but significant increases in levels of total tau (t-tau) and tau phosphorylated at threonine-181 (p-tau) (*p* = 0.02 and *p* = 0.01 respectively).

**Table 1:** Demographics and CSF biomarker profiles of sCAA patients and control subjects in the PEA exploration study

	CON	sCAA	*p*-value
**# patients (n)**	50	34	-
**Age (y)**	71.8 (68.5–74.9)	73.4 (68.2–77.2)	*p* = 0.84 (ns)^a^
**Sex M/F (%M)**	25/25 (50%)	18/16 (53%)	*p* = 0.79 (ns)^c^
**MoCA** ^**d**^	28 (26.5–29)	24 (21–26)	***p*** < 0.0001 (****) ^**b**^
**CSF biomarkers**			
**Aβ40 (pg/mL)**	11760 (8961–14736)	7592 (6337–9037)	*p* < 0.0001 (****) ^**a**^
**Aβ42 (pg/mL)**	914 (565–1238)	353 (289–425)	***p*** < 0.0001 (****) ^**b**^
**t-tau (pg/mL)**	327 (233–485)	412 (280–632)	***p*** = 0.02 (*) ^**b**^
**p-tau (pg/mL)**	39.7 (30.1–55.5)	55.4 (35.9–76.3)	***p*** = 0.01 (*) ^**b**^

Data is presented as median (interquartile range). ^a^ Student’s t-test, ^b^ Mann-Whitney U test, ^c^ Chi-square, ^d^ scoring available for respectively 25 (controls) and 28 (sCAA) participants. MoCA = Montreal Cognitive Assessment, t-tau = total tau, p-tau = tau phosphorylated at threonine 181, **** *p* 0.0001, * *p* ≤ 0.05, ns = non-significant

### Subject selection for ELLA NFL analysis

For validation purposes, NFL was analysed in CSF and serum of 38 sCAA patients (all BIONIC, including 10 samples that were not subjected to PEA analysis) and 26 controls (all CAFE, including 1 sample that was not subjected to PEA analysis). The resulting groups were matched for age (*p* = 0.99) and sex (*p* = 0.92). Characteristics of the participants in the validation group are shown in Table 2.

**Table 2:** Demographics and CSF/serum biomarker levels of sCAA patients and control subjects in the validation study

	CON	sCAA	*p*-value
**# patients** (n)	26	38	-
**Age** (y)	71.4 (69.2–74.9)	72.9 (67.3–75.8)	*p* = 0.99 (ns)^a^
**Sex M/F** (%M)	14/12 (54%)	19/18 (51%)	*p* = 0.92 (ns)^c^
**MoCA**	28 (27–29)	24.5 (21–27)	***p*** = 0.0002 (***) ^**b**^
**CSF/serum biomarker levels**			
**CSF Aβ40** (pg/mL)	12,713 (10,782–15,387)	7901 (6553–9427)	***p*** < 0.0001 (****) ^**a**^
**CSF Aβ42** (pg/mL)	1116 (828–1348)	367 (288–457)	***p*** < 0.0001 (****) ^**b**^
**CSF t-tau** (pg/mL)	356 (277–485)	428 (284–572)	*p* = 0.10 (ns)
**CSF p-tau** (pg/mL)	41.3 (33.3–59.5)	55.9 (35.9–71.6)	*p* = 0.08 (ns)
**CSF ELLA NFL** (pg/mL)	1306 (806–1482)	2284 (1526–3464)	***p*** < 0.0001 (****) ^**b**^
**Serum ELLA NFL** (pg/mL)	28.8 (24.9–33.7)	43.3 (31.7–66.8)	***p*** < 0.0001 (****) ^**b**^
**Q**_**NFL**_ (CSF NFL/serum NFL)	39.1 (30.5–49.3)	51.3 (42.2–74.2)	***p*** = 0.002 (**) ^**b**^
**Q**_**ALB**_ (CSF albumin / serum albumin *10E-3)	6.6 (5.5–7.8)	6.2 (5.3–8.2)	*p* = 0.88 (ns)

^a^ Student’s t-test, ^b^ Mann-Whitney U test, ^c^ Chi-square. MoCA = Montreal Cognitive Assessment, Aβ = amyloid beta, t-tau = total tau, p-tau = tau phosphorylated at threonine 181, NFL = neurofilament light chain, ALB = albumin **** *p* < 0.0001, *** *p* < 0.001, ** *p* ≤ 0.01, ns = non-significant

### CSF and serum analysis of amyloid β, tau and albumin

CSF was analysed for AD CSF biomarkers Aβ40, Aβ42, t-tau, and p-tau, all measured using a Lumipulse chemiluminescent assay (Fujirebio, Belgium). CSF (5x diluted) and serum albumin (400x diluted) were determined using an Atellica NEPH 630 nephelometric assay (Siemens Healthineers, Erlangen, Germany).

### Proximity extension assay

A multiplex PEA was performed using the Olink® Explore 384 Neurology panel (Olink, Uppsala, Sweden). This assay consists of 367 neurology-associated proteins (full list can be retrieved from https://olink.com/products-services/explore/). Data was expressed as normalised protein expression (NPX) values. NPX values are relative expression values which have been log2 transformed to normalize data and to minimize intra- and inter-assay variation. Analytes were included for further analysis if signals exceeded the limit of detection (LoD) for ≥ 70% of samples of the sCAA and/or control groups. Fold-changes of expression levels were examined by 2^(ΔNPX)^, in which ΔNPX is defined as median NPX_sCAA_ – NPX_CON_ values.

### ELLA NFL assay

An ELLA automated immunoassay system (Biotechne, Minneapolis, MN, USA), was used to analyse human CSF and serum NFL levels. In this assay, 25 µL of CSF or serum was diluted twofold with reagent diluent to a total volume of 50 µL prior to pipetting the solution in the analysis cartridge. The assay ran automated analyses in technical triplicate.

### Data analysis

Statistical analysis was performed using Graphpad Prism 9.5.0 (Graphpad Software, USA) and RStudio v.2022.02.1. Statistical significant differences were defined at *p* ≤ 0.05 (*), *p* ≤ 0.01 (**), *p* ≤ 0.001 (***), and *p* < 0.0001 (****). (Non-)parametric data were assessed using Student’s t-tests and Mann-Whitney U tests respectively. Spearman correlation analyses were performed to assess associations between variables. Logarithmic regression analysis was performed to assess the relationship between CSF and serum NFL levels in subjects. The ratios of CSF to serum NFL were computed as Q_NFL_ levels. This method was also applied to calculate the Q_ALB_ (ratio of CSF and serum albumin levels*10E-3). Receiver operating characteristic (ROC) curves were constructed to determine the ability of biomarkers to differentiate between sCAA patients and controls. ROC curves were compared using DeLong’s test [15].

## Results

**Fig. 1:**
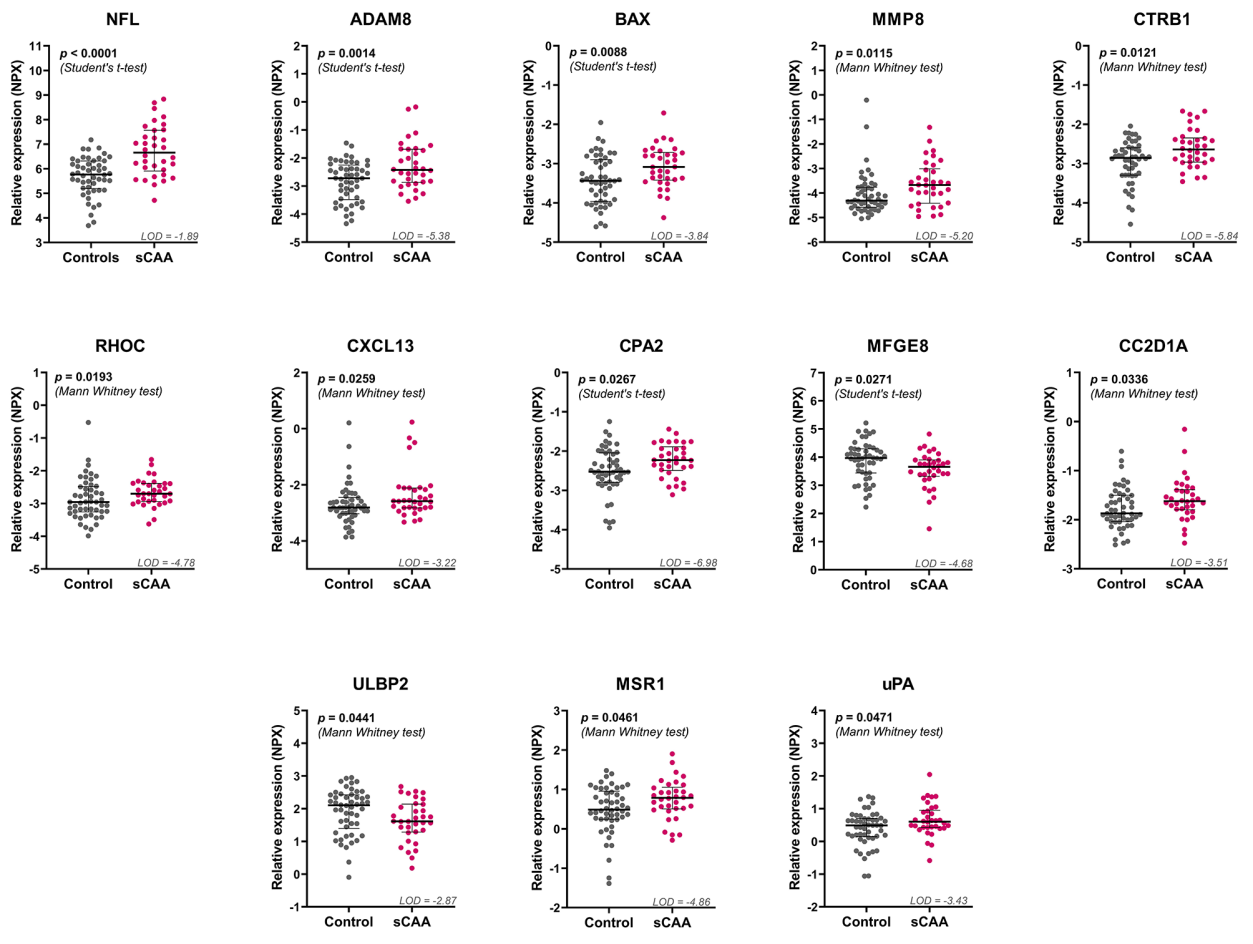
Scatter plots of proximity extension assay (PEA) biomarker candidates in CSF of sCAA patients compared to controls. PEA analysis in CSF of sCAA patients and controls produced 13 significantly differential protein biomarkers. Statistical testing was performed using Student’s t-tests (parametric data) and Mann-Whitney U tests (non-parametric data). The LoD of each protein is indicated in the graphs and presented as dashed line (in case the LoD was within the plotted y-axis range)

**Fig. 2:**
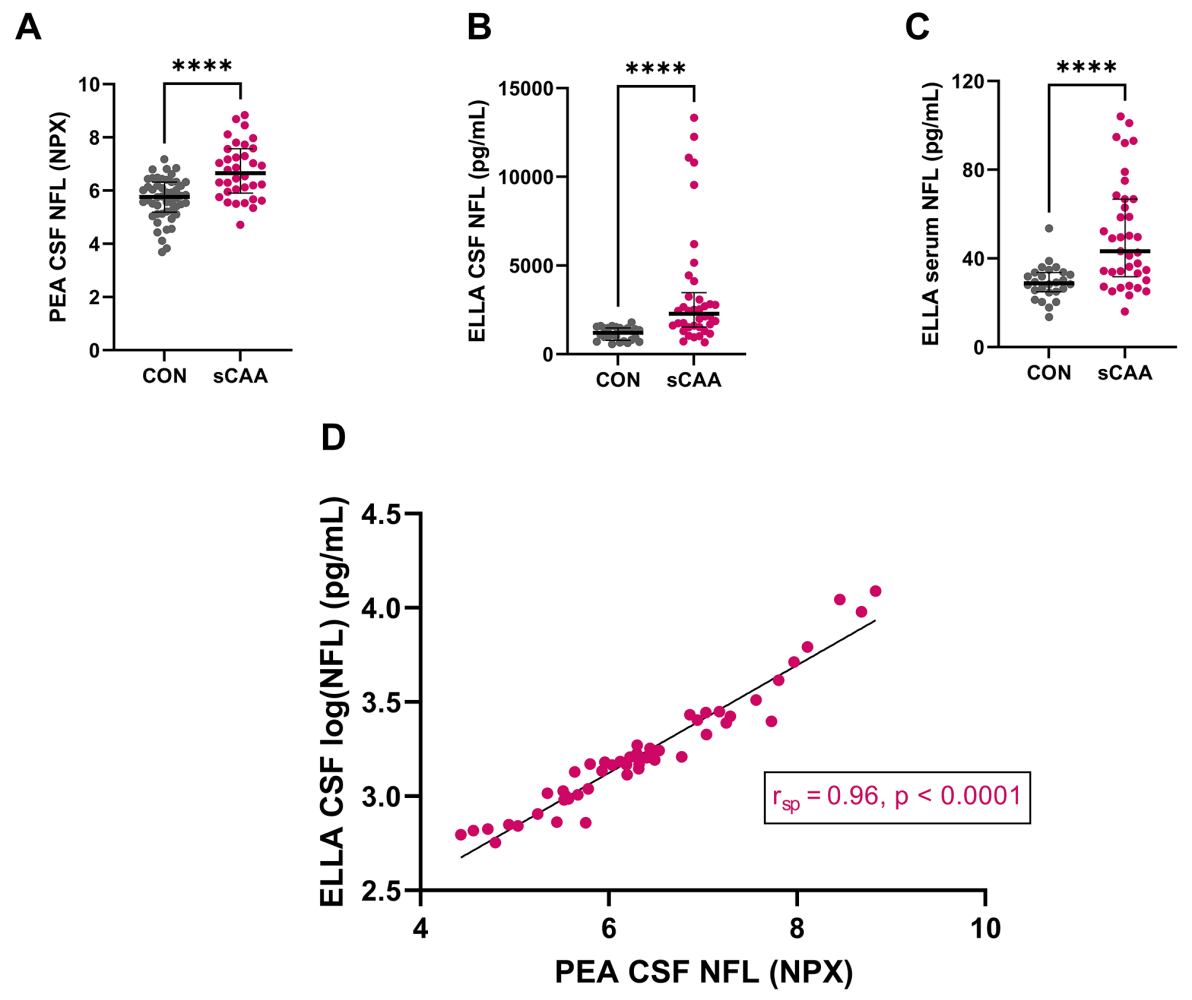
Dot and scatter plots of CSF and serum NFL measurements. (A) Dot plot of PEA CSF NFL measurement in controls and sCAA patients. (**B**) Dot plot of ELLA CSF NFL measurement in controls and sCAA patients. (**C**) Dot plot of ELLA serum NFL measurement in controls and sCAA patients. (**D**) Scatter plot of PEA CSF NFL measurements against log-transformed ELLA NFL measurements in CSF. R_SP_ = Spearman correlation. **** *p* < 0.0001

Of the 367 proteins included in the PEA exploration panel, 263 (72%) presented expression levels greater than respective LoD in > 70% of either sCAA and/or control groups. Of these 263, 13 proteins presented differential expression levels between sCAA patients and control subjects Table 3; Fig. 1. In descending order of significance: neurofilament light chain (NFL; *p* < 0.0001) Fig. 2A, a disintegrin with metalloproteinase domain-containing protein 8 (ADAM8; *p* = 0.001), apoptosis regulator BAX (BAX; *p* = 0.009), matrix metalloproteinase-8 (MMP8; *p* = 0.01), chymotrypsinogen B1 (CTRB1; *p* = 0.01), Ras homolog gene family, member C (RHOC; *p* = 0.02), chemokine (C-X-C motif) ligand 13 (CXCL13; *p* = 0.03), carboxypeptidase A2 (CPA2; *p* = 0.03), milk fat globule-EGF factor 8 (MFGE8; *p* = 0.03), coiled-coil and C2 domain-containing protein A1 (CC2D1A; *p* = 0.03), UL16-binding protein 2 (ULBP2; *p* = 0.04), macrophage scavenger receptor 1 (MSR; *p* = 0.05), and urokinase plasminogen activator (uPA; *p* = 0.05). For two of these proteins, BAX and CXCL13, respectively 19% and 14% of the data points collected were lower than the LoD.

**Table 3:** Overview of proteins which were significantly different between sCAA patients and control subjects

Protein	Gene	CON NPX	sCAA NPX	FC sCAA/CON	*P*-value
Neurofilament light chain	*Nfel*	5.77 (5.19 : 6.32)	6.66 (5.91 : 7.57)	1.85	***p*** < 0.0001 (****) ^**a**^
ADAM metallopeptidase domain 8.	*Adam8*	-2.72 (-3.48 : -2.25)	-2.43 (-2.87 : -1.69)	1.23	***p*** = 0.001 (***) ^**a**^
Apoptosis regulator BAX	*Bax*	-3.44 (-3.96 : -2.90)	-3.09 (-3.42 : -2.72)	1.28	*p* = 0.009 (**) ^**a**^
Matrix metalloproteinase-8	*Mmp8*	-4.31 (-4.58 : -3.76)	-3.67 (-4.42 : -3.01)	1.55	***p*** = 0.01 (*) ^**b**^
Chymotrypsinogen B1	*Ctrb1*	-2.85 (-3.30 : -2.60)	-2.64 (-2.96 : -2.35)	1.16	***p*** = 0.01 (*) ^**b**^
Ras homolog gene family, member C	*Rhoc*	-2.96 (-3.26 : -2.49)	-2.70 (-2.94 : -2.70)	1.19	***p*** = 0.02 (*) ^**b**^
Chemokine (C-X-C motif) ligand 13	*Cxcl13*	-2.81 (-3.03 : -2.45)	-2.58 (-2.82 : -2.11)	1.17	***p*** = 0.03 (*) ^**b**^
Carboxypeptidase A2	*Cpa2*	-2.53 (-2.80 : -2.05)	-2.23 (-2.50 : -1.89)	1.23	***p*** = 0.03 (*) ^**a**^
Milk fat globule-EGF factor 8 protein	*Mfge8*	3.97 (3.44 : 4.29)	3.66 (3.32 : 3.90)	0.81	***p*** = 0.03 (*) ^**a**^
Coiled-coil and C2 domain-containing protein 1 A	*Cc2d1a*	-1.87 (-2.04 : -1.50)	-1.62 (-1.80 : -1.39)	1.19	***p*** = 0.03 (*) ^**b**^
UL16-binding protein 2	*Ulbp2*	2.11 (1.40 : 2.42)	1.61 (1.29 : 2.14)	0.71	***p*** = 0.04 (*) ^**b**^
Macrophage scavenger receptor 1	*Msr1*	0.49 (0.24 : 0.96)	0.79 (0.51 : 1.06)	1.23	***p*** = 0.05 (*) ^**b**^
Urokinase plasminogen activator	*Plau*	0.49 (0.15 : 0.70)	0.60 (0.42 : 0.95)	1.08	***p*** = 0.05 (*) ^**b**^

Relative NPX values of sCAA patients and controls (CON) are displayed as median with interquartile range (IQR). Fold-change (FC) was calculated using the formular FC = 2^ΔNPX^. ^a^ Student’s t-test, ^b^ Mann-Whitney U test. **** *p* < 0.0001, *** *p* < 0.001, ** *p* < 0.01, * *p* < 0.05

Correlation analyses between the 13 differentially expressed proteins, and clinical and imaging sCAA measures (age of participants, score on the MOCA, presence of ICH, number of CMB, presence/extent of cSS, CAA SVD) revealed significant associations (Figure S1). Eight proteins significantly correlated with age (NFL, MMP8, RHOC, CXCL13, CPA2, CC2D1A, MSR1, uPA). MoCA scores significantly correlated with ULBP2 (r_sp_ = 0.39; *p* = 0.004), and NFL (r_sp_ = -0.37; *p* = 0.006). Positive correlations with the number of CMBs existed for NFL (r_sp_ = 0.44; *p* = 0.001), ADAM8 (r_sp_ = 0.40; *p* = 0.005), and MMP8 (r_sp_ = 0.38; *p* = 0.007), whereas MFGE8 displayed a negative correlation (r_sp_ = -0.46; *p* = 0.0008). The degree of cSS correlated positively with NFL (r_sp_ = 0.35; *p* = 0.002). Lastly, NFL (r_sp_ = 0.54; *p* < 0.0001) and MFGE8 (r_sp_ = -0.45; *p* = 0.001) correlated significantly with the CAA SVD-burden score.

ELLA analyses revealed significant elevations of CSF NFL levels in sCAA (median 2284 pg/mL) as compared to controls (median: 1306 pg/mL) (*p* < 0.0001) (Table 2; Fig. 2B). Similarly, serum NFL levels were significantly elevated in sCAA (median: 43.3 pg/mL) vs. controls (28.8 pg/mL; *p* < 0.0001) (Table 2; Fig. 2C). Correlation analyses of the PEA NPX.

NFL values and log-transformed ELLA CSF NFL results showed a strong degree of correlation (r_sp_= 0.96, *p* < 0.0001) (Fig. 2D).

**Fig. 3:**
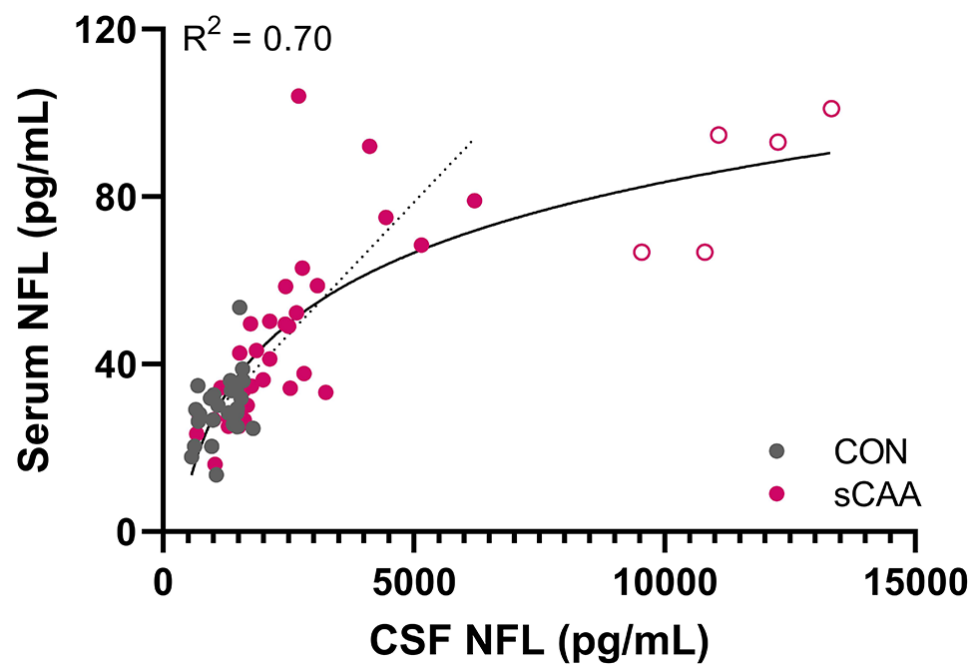
Scatter plots on the relationship between CSF and serum ELLA NFL measurements. Scatter plots of CSF and serum NFL levels, in control subjects and sCAA patients. The lower end of the graph shows a relatively linear relationship (irrespective of CAA pathology) between CSF and serum NFL levels. However, a non-linear relationship appears between CSF and serum NFL levels, with higher CSF NFL levels (y = -141.2 + 56.2*log(x)). Solid line displays logarithmic regression through all data points, dashed line is linear regression (constructed excluding outliers, which are indicated as open dots)

Whereas at lower CSF NFL levels there appeared to be a directly proportional relationship between CSF and serum NFL levels, this was not the case at higher CSF NFL levels. An apparent logarithmic relationship existed between CSF NFL and serum NFL concentrations over the full concentration range, with an R^2^ of 0.70. (Figure 3).

Correlation analyses between ELLA CSF NPX levels and available clinical and imaging measures revealed identical patterns to the associations observed for PEA NFL levels (Figure S2): a negative significant correlation was found between CSF NFL levels and MOCA scores (r_sp_ = -0.37; *p* = 0.003). Positive, significant correlations were discovered between CSF NFL levels and the number of CMBs (r_sp_ = 0.48; *p* < 0.0001), the degree of cSS (r_sp_ = 0.42; *p* = 0.0006), and the CAA SVD-burden score (r_sp_ = 0.45; *p* = 0.0002) respectively. Contrasting with the exploration study, no significant correlation between CSF NFL and age of participants was found in the validation study.

**Fig. 4:**
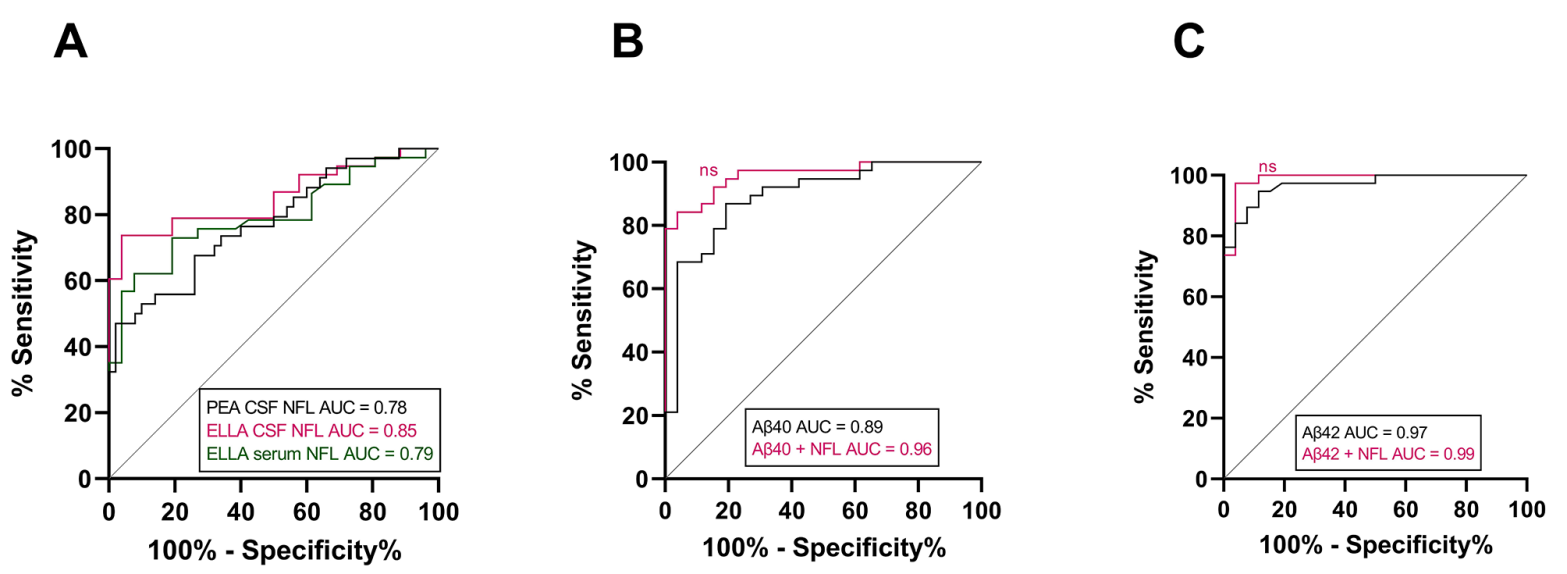
Receiver operating characteristic (ROC) curves on the discrimination ability between sCAA patients and controls. (A) ROC curves of PEA CSF, ELLA CSF and ELLA serum NFL levels of sCAA and controls. (**B**) ROC curves of CSF Aβ40 and CSF Aβ40 + ELLA NFL of sCAA and controls. Aβ40 + NFL did not show improved discrimination performance compared to Aβ40 (*p* = 1.00). (**C**) ROC curves of CSF Aβ42 and CSF Aβ42 + ELLA NFL of sCAA and controls. No improved performance was observed for Aβ42 + NFL compared to Aβ42 (*p* = 1.00). AUC = area under the curve. ns not significant

ROC curves revealed good separation between sCAA patients and control subjects using PEA CSF NFL (AUC = 0.78), ELLA CSF NFL (AUC = 0.85) and serum NFL levels (AUC = 0.79) (Fig. 4A). Combinations of PEA differentially expressed proteins did not provide better discrimination than NFL between sCAA and controls [data not shown]. Aβ40 demonstrated good discrimination ability between sCAA patients and controls (AUC = 0.89) (Fig. 4B). The combination of NFL with Aβ40 did not significantly improve discrimination ability compared to Aβ40, between sCAA patients and control subjects (AUC = 0.96, *p* = 1.00). The ROC curve for Aβ42 showed good discrimination performance (AUC = 0.97), but this did not improve for Aβ42 + NFL (AUC = 0.99, *p* = 1.00) (Fig. 4C).

**Fig. 5:**
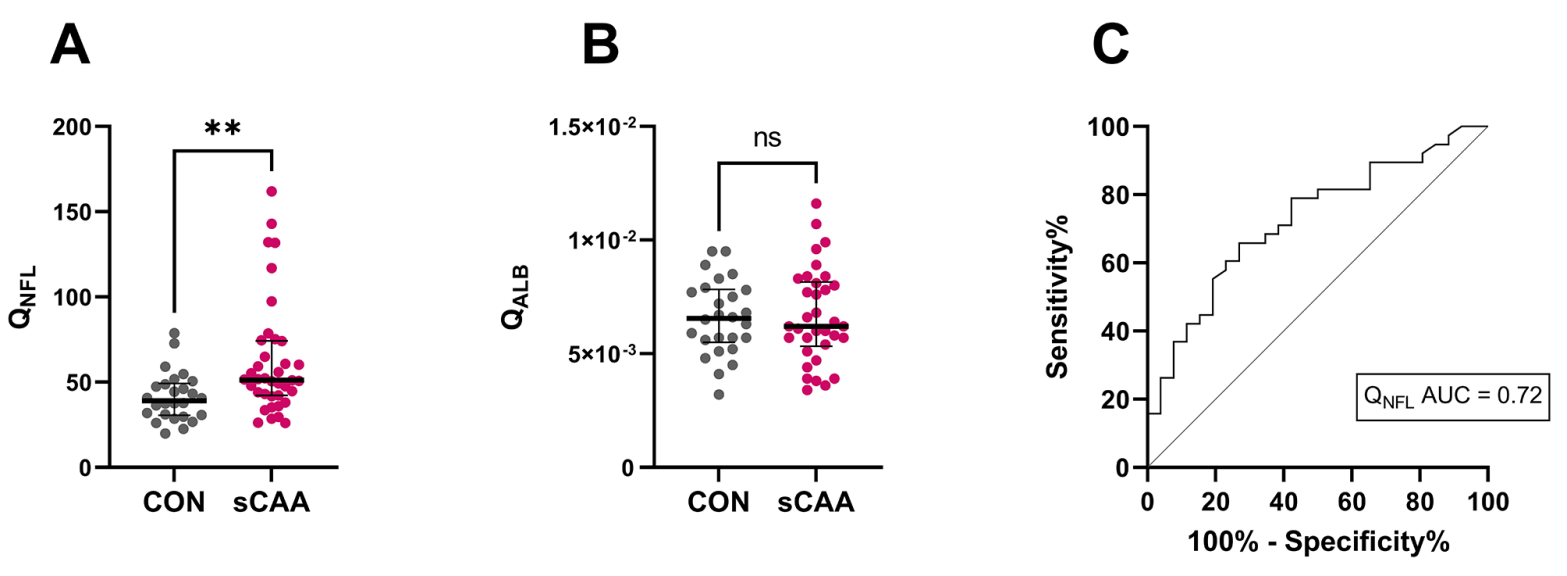
Dot and scatter plots on Q_NFL_and Q_**ALB**_. (**A**) Dot plot of Q_NFL_ in controls and sCAA patients. (**B**) Dot plot of Q_ALB_ in controls and sCAA patients. (**C**) ROC curve of Q_NFL_ in discriminating controls from sCAA patients. AUC = area under the curve, ** *p* ≤ 0.01, ns not significant

CSF: serum NFL ratios (Q_NFL_) were significantly increased in sCAA patients compared to control subjects (*p* = 0.004) (Fig. 5A). Q_ALB_ levels did not differ significantly between sCAA patients (median: 6.6) and controls (median: 6.2, *p* = 0.88) (Fig. 5B). ROC analysis of the Q_NFL_ discriminated CAA patients from control subjects with an AUC of 0.72 (Fig. 5C).

## Discussion

This multiplex biomarker discovery study identified multiple emerging CSF biomarker candidates for differentiation of sCAA from controls. We were able to identify NFL as biomarker in CSF and serum of sCAA patients compared to controls employing two independent techniques (PEA and ELLA).

NFL is one of the subunits of neurofilaments and plays a crucial role in maintaining the integrity of neuronal axons [16]. NFL has previously been reported to be elevated in plasma and CSF of sCAA patients compared to controls [17, 18]. NFL is a well-known serum/plasma/CSF biomarker of axonal damage in multiple forms of neurodegeneration (including multiple sclerosis, atypical parkinsonisms, frontotemporal dementia, amyotrophic lateral sclerosis, and prion diseases) [19–22]. NFL elevations are often associated with generalized neurodegeneration, rather than specifically with sCAA, which might limit its potential as a diagnostic biomarker for sCAA.

We found a large degree of correlation between measurements using PEA and the (log-transformed) ELLA NFL assay concerning the CSF analyses, which reinforces trueness of measurements, and in turn, increased the robustness and reproducibility of results. Earlier studies already described a similar, very high degree of correlation between NFL measurements on automated ELLA and Simoa assays [23].

The relationship between CSF and serum NFL seemed to follow a non-linear pattern across the entire concentration range: whereas at low levels CSF NFL appeared to proportionally correlate to serum NFL, higher levels of CSF NFL seemed not to be proportionally reflected in equally higher serum NFL levels in our study. This might be considered as negative implications for the potential use of serum NFL as a monitoring or prognostic biomarker. Such a potential non-linear relationship at high CSF NFL concentrations between CSF and serum NFL could also be noted in studies of other neurodegenerative diseases, such as Parkinson’s disease [22], but was absent in other studies [24].

Additionally, Q_NFL_ was increased in sCAA compared to controls. The elevated Q_NFL_ suggests that in sCAA patients, NFL was present in CSF in a relatively higher proportion, compared to controls. We did not observe similar differences in Q_ALB_ between sCAA patients and controls. This could imply that the integrity of the blood-CSF barrier (for which Q_ALB_ is a proxy) remains unaltered and does not explain these observed differences in the Q_NFL_. This would indicate that increased CSF NFL is not proportionally exchanged with serum, possibly through limited exchange of CSF NFL to blood in the arachnoid villi, or the possibility of dose-dependent degradation of NFL in the circulation.

CSF NFL levels were positively associated with neurovascular damage in the form of numbers of CMBs and cSS. Additionally, higher CSF NFL levels negatively correlated with MOCA scores. This suggests that neurovascular sCAA pathology incites and/or aggravates neurodegeneration, which in turn appears to have repercussions for the clinical presentation of sCAA patients, in the form of increased degrees of cognitive impairment.

Among the significantly different biomarkers that we identified, two confirmed results previously obtained by our team in comparable CSF immunoassay studies: milk fat globule-EGF 8 (MFGE8) and urokinase plasminogen activator (uPA) [11, 25]. MFGE8 (also known as lactadherin) is a secreted, extracellular matrix protein and is thought to contribute to a great variety of molecular and cellular interactions, including cellular adhesion and inhibition of coagulation [26]. Full length MFGE8 has also been investigated in the context of CAA pathology; it was found that it was overexpressed in CAA vessels, colocalizes with vascular Aβ deposits, and is decreased in CSF of CAA patients, compared to both AD and controls [25]. A small, 50 amino acid polypeptide fragment of MFGE8 (medin) also co-localized with vascular Aβ, and higher vascular MGFE8 expression levels have been associated with an increased degree of cognitive decline in AD [27]. uPA has a major role in the activation of plasminogen into plasmin, an important process in the initiation of thrombolysis. uPA was overexpressed by vascular smooth muscle cells under stimulation of APP [28]. Additionally, uPA was overexpressed in vascular tissue and CSF of transgenic *APP* rodent models for CAA and in sCAA patients [11]. Sidenote is that the sCAA population in this study in small part (*n* = 9) overlapped with the results in our PEA study, which might have influenced results.

In our study, we also uncovered significant differences in the expression of other biomarker candidates, such as ADAM8, MMP8, and MSR1 (all upregulated). ADAM8 has been implied to function as an alpha-secretase, involved in non-pathological processing of APP [29, 30]. Our observation of increased expression of ADAM8 in CSF of sCAA patients could point to disruption of physiologic processing of APP. MMP8 has to our knowledge not been studied in relation to CAA pathology [31]. However, in general, matrix metalloproteinases and their inhibitors appear to be involved in CAA pathology [10, 32]. Lastly, MSR1 is known to facilitate microglial phagocytosis of Aβ aggregates [33, 34]. MSR1-knockout mice have shown increased vascular amyloid pathology and decreased clearance rates of vascular amyloid, compared to wild-type mice [35]. Additionally, another similar PEA study on CSF and plasma of AD patients revealed a negative correlation between CSF MSR1 levels and severity of AD pathology ranging from healthy controls to MCI and to AD [36]. Potential explanations for these contrasting results could be found in different study designs, and differential activation and affinity patterns of microglia for either vascular or parenchymal amyloid pathology. Other proteins which were found to be significantly differentially expressed in sCAA patients compared to control subjects (BAX, CC2D1A, CPA2, CTRB1, CXCL13, RHOC, ULBP2) have (to our knowledge) not previously been associated with CAA, AD or amyloidotic diseases in general, which warrants further research into their biomarker potential.

Strengths of our study include the use of cohorts of sCAA patients and control subjects which have been very well characterized clinically. Additionally, the replication of results using orthogonal analytical techniques for three biomarker candidates (NFL, MFGE8 [25] and uPA [11]) support the robustness of our findings. Lastly, the sizes of sCAA and control groups studied are relatively large, compared to most other CSF biomarker studies on sCAA in literature [10, 11, 26, 37, 38]. Weaknesses of this study include a partial overlap of patients with sCAA in study populations. Also, another weakness is the fact that in clinical practice, sCAA will often have to be differentiated from other neurological diseases, instead of differentiation between patients with sCAA and healthy controls. The absence of study populations with other neurological diseases (e.g. AD) in our study likely limited our assessment of the diagnostic potential of NFL as a biomarker for sCAA. In addition, the diagnostic value of NFL is limited since increased levels of NFL are observed in many disorders associated with neurodegeneration. It would be interesting, though, to longitudinally study (CSF or serum) NFL to assess its potential as a possible biomarker of disease progression in CAA. Lastly, the lack of independent validation of identified biomarker candidates other than NFL, MFGE8 and uPA is also a limitation of our study [39]. This predisposes results to the false-positive identification of proteins as potential biomarkers and incentivises the need of validation of biomarker candidates in independent cohorts, or using independent analytical techniques. Therefore, further research will have to confirm or reject the biomarker potential of the remaining 10 CSF biomarker candidates.

In conclusion, our results show that screening PEA analyses are able to identify candidate biomarkers for CAA, which can be validated through use of orthogonal validation efforts. Additionally, NFL appears to be a very effective biomarker to distinguish sCAA patients from controls, but might have limited specificity for sCAA, due to its broader associations with more generalised neurodegeneration.

### Electronic supplementary material

Below is the link to the electronic supplementary material.

Supplementary Material 1

## Data Availability

The datasets used and/or analysed during the current study are available from the corresponding author on reasonable request.

## References

[1] Gatti L, Tinelli F, Scelzo E, Arioli F, Di Fede G, Obici L (2020). Understanding the pathophysiology of cerebral amyloid Angiopathy. Int J Mol Sci.

[2] Thal DR, Ghebremedhin E, Rüb U, Yamaguchi H, Del Tredici K, Braak H (2002). Two types of sporadic cerebral amyloid angiopathy. J Neuropathol Exp Neurol.

[3] Greenberg SM, Bacskai BJ, Hernandez-Guillamon M, Pruzin J, Sperling R, van Veluw SJ (2020). Cerebral amyloid angiopathy and Alzheimer disease — one peptide, two pathways. Nat Reviews Neurol.

[4] Charidimou A, Boulouis G, Gurol ME, Ayata C, Bacskai BJ, Frosch MP (2017). Emerging concepts in sporadic cerebral amyloid angiopathy. Brain.

[5] Koemans EA, Chhatwal JP, van Veluw SJ, van Etten ES, van Osch MJP, van Walderveen MAA (2023). Progression of cerebral amyloid angiopathy: a pathophysiological framework. Lancet Neurol.

[6] Greenberg SM, Charidimou A (2018). Diagnosis of cerebral amyloid Angiopathy. Stroke.

[7] Sancesario G, Bernardini S (2019). AD biomarker discovery in CSF and in alternative matrices. Clin Biochem.

[8] Hondius DC, Eigenhuis KN, Morrema THJ, van der Schors RC, van Nierop P, Bugiani M (2018). Proteomics analysis identifies new markers associated with capillary cerebral amyloid angiopathy in Alzheimer’s disease. Acta Neuropathol Commun.

[9] van den Berg E, Nilsson J, Kersten I, Brinkmalm G, de Kort AM, Klijn CJM (2023). Cerebrospinal Fluid Panel of Synaptic Proteins in cerebral amyloid angiopathy and Alzheimer’s Disease. J Alzheimers Dis.

[10] Vervuurt M, de Kort AM, Jäkel L, Kersten I, Abdo WF, Schreuder F (2023). Decreased ratios of matrix metalloproteinases to tissue-type inhibitors in cerebrospinal fluid in sporadic and hereditary cerebral amyloid angiopathy. Alzheimers Res Ther.

[11] Vervuurt M, Zhu X, Schrader J, de Kort AM, Marques TM, Kersten I (2022). Elevated expression of urokinase plasminogen activator in rodent models and patients with cerebral amyloid angiopathy. Neuropathol Appl Neurobiol.

[12] De Kort AM, Kuiperij HB, Alcolea D, Kersten I, Versleijen AAM, Greenberg SM (2021). Cerebrospinal fluid levels of the neurotrophic factor neuroleukin are increased in early Alzheimer’s disease, but not in cerebral amyloid angiopathy. Alzheimers Res Ther.

[13] De Kort AM, Kuiperij HB, Marques TM, Jäkel L, van den Berg E, Kersten I (2023). Decreased cerebrospinal fluid amyloid β 38, 40, 42, and 43 levels in sporadic and Hereditary Cerebral amyloid Angiopathy. Ann Neurol.

[14] Linn J, Halpin A, Demaerel P, Ruhland J, Giese AD, Dichgans M (2010). Prevalence of superficial siderosis in patients with cerebral amyloid angiopathy. Neurology.

[15] DeLong ER, DeLong DM, Clarke-Pearson DL (1988). Comparing the areas under two or more correlated receiver operating characteristic curves: a nonparametric approach. Biometrics.

[16] Yuan A, Rao MV, Veeranna, Nixon RA (2012). Neurofilaments at a glance. J Cell Sci.

[17] Banerjee G, Ambler G, Keshavan A, Paterson RW, Foiani MS, Toombs J (2020). Cerebrospinal fluid biomarkers in cerebral amyloid Angiopathy. J Alzheimers Dis.

[18] McCarter SJ, Lesnick TG, Lowe VJ, Rabinstein AA, Przybelski SA, Algeciras-Schimnich A (2022). Association between Plasma Biomarkers of Amyloid, Tau, and neurodegeneration with cerebral microbleeds. J Alzheimers Dis.

[19] Gaetani L, Blennow K, Calabresi P, Filippo MD, Parnetti L, Zetterberg H (2019). Neurofilament light chain as a biomarker in neurological disorders. J Neurol Neurosurg Psychiatry.

[20] Delaby C, Alcolea D, Carmona-Iragui M, Illán-Gala I, Morenas-Rodríguez E, Barroeta I (2020). Differential levels of neurofilament light protein in cerebrospinal fluid in patients with a wide range of neurodegenerative disorders. Sci Rep.

[21] Sferruzza G, Bosco L, Falzone YM, Russo T, Domi T, Quattrini A (2022). Neurofilament light chain as a biological marker for amyotrophic lateral sclerosis: a meta-analysis study. Amyotroph Lateral Scler Frontotemporal Degeneration.

[22] Marques TM, Rumund Av, Oeckl P, Kuiperij HB, Esselink RAJ, Bloem BR (2019). Serum NFL discriminates Parkinson disease from atypical parkinsonisms. Neurology.

[23] Truffi M, Garofalo M, Ricciardi A, Cotta Ramusino M, Perini G, Scaranzin S (2023). Neurofilament-light chain quantification by Simoa and Ella in plasma from patients with dementia: a comparative study. Sci Rep.

[24] Kläppe U, Sennfält S, Lovik A, Finn A, Bofaisal U, Zetterberg H (2024). Neurodegenerative biomarkers outperform neuroinflammatory biomarkers in amyotrophic lateral sclerosis. Amyotroph Lateral Scler Frontotemporal Degeneration.

[25] Marazuela P, Solé M, Bonaterra-Pastra A, Pizarro J, Camacho J, Martínez-Sáez E (2021). MFG-E8 (Lactadherin): a novel marker associated with cerebral amyloid angiopathy. Acta Neuropathol Commun.

[26] Kamińska A, Enguita FJ, Stępień EŁ (2018). Lactadherin: an unappreciated haemostasis regulator and potential therapeutic agent. Vascul Pharmacol.

[27] Wagner J, Degenhardt K, Veit M, Louros N, Konstantoulea K, Skodras A (2022). Medin co-aggregates with vascular amyloid-β in Alzheimer’s disease. Nature.

[28] Davis J, Wagner MR, Zhang W, Xu F, Van Nostrand WE (2003). Amyloid beta-protein stimulates the expression of urokinase-type plasminogen activator (uPA) and its receptor (uPAR) in human cerebrovascular smooth muscle cells. J Biol Chem.

[29] Naus S, Reipschläger S, Wildeboer D, Lichtenthaler SF, Mitterreiter S, Guan Z (2006). Identification of candidate substrates for ectodomain shedding by the metalloprotease-disintegrin ADAM8. Biol Chem.

[30] Amour A, Knight CG, English WR, Webster A, Slocombe PM, Knäuper V (2002). The enzymatic activity of ADAM8 and ADAM9 is not regulated by TIMPs. FEBS Lett.

[31] Lee E-J, Han JE, Woo M-S, Shin JA, Park E-M, Kang JL (2014). Matrix Metalloproteinase-8 plays a pivotal role in Neuroinflammation by modulating TNF-α activation. J Immunol.

[32] Jäkel L, Kuiperij HB, Gerding LP, Custers EEM, van den Berg E, Jolink WMT (2020). Disturbed balance in the expression of MMP9 and TIMP3 in cerebral amyloid angiopathy-related intracerebral haemorrhage. Acta Neuropathol Commun.

[33] Paresce DM, Ghosh RN, Maxfield FR (1996). Microglial Cells Internalize Aggregates of the Alzheimer’s disease amyloid β-Protein Via a scavenger receptor. Neuron.

[34] Chung H, Brazil MI, Irizarry MC, Hyman BT, Maxfield FR (2001). Uptake of fibrillar β-amyloid by microglia isolated from MSR-A (type I and type II) knockout mice. NeuroReport.

[35] Lifshitz V, Weiss R, Levy H, Frenkel D (2013). Scavenger receptor A Deficiency accelerates cerebrovascular amyloidosis in an animal model. J Mol Neurosci.

[36] Miron J, Picard C, Labonté A, Auld D, Poirier J (2022). For the P-ADrg. MSR1 and NEP are correlated with Alzheimer’s disease amyloid Pathology and Apolipoprotein alterations. J Alzheimers Dis.

[37] Kuiperij HB, Hondius DC, Kersten I, Versleijen AAM, Rozemuller AJM, Greenberg SM (2020). Apolipoprotein D: a potential biomarker for cerebral amyloid angiopathy. Neuropathol Appl Neurobiol.

[38] De Kort AM, Kuiperij HB, Kersten I, Versleijen AAM, Schreuder FHBM, Van Nostrand WE (2022). Normal cerebrospinal fluid concentrations of PDGFRβ in patients with cerebral amyloid angiopathy and Alzheimer’s disease. Alzheimer’s Dement.

[39] Hernández B, Parnell A, Pennington SR (2014). Why have so few proteomic biomarkers survived validation? (sample size and independent validation considerations). Proteomics.

