# Supplementary material for: Proximity extension assay in cerebrospinal fluid identifies neurofilament light chain as biomarker of neurodegeneration in sporadic cerebral amyloid angiopathy
Source: Alzheimers Res Ther. 2024 May 14;16:108. doi: 10.1186/s13195-024-01473-0 (PMC11092079; doi:10.1186/s13195-024-01473-0)

**Supplementary material**

**Proximity extension assay biomarker discovery in cerebrospinal fluid reveals NFL as biomarker for cerebral amyloid angiopathy**

Marc Vervuurt MSc^1#^; H. Bea Kuiperij PhD^1#^; Anna M. de Kort MSc, MD^1^; Iris Kersten BSc.^1^; Catharina J. M. Klijn MD, PhD^1^; Floris H. B. M. Schreuder MD, PhD^1^; Marcel M. Verbeek PhD^1,2^

^1^ Radboud University Medical Center, Donders Institute for Brain, Cognition and Behaviour, Department of Neurology, Nijmegen, The Netherlands

^2^ Radboud University Medical Center, Department of Human Genetics, Nijmegen, The Netherlands

#These authors equally contributed to this article.

*Correspondence to: Dr. Marcel M. Verbeek, Department of Human Genetics, 830 TML, Radboud University Medical Center, P.O. Box 9101, 6500 HB Nijmegen, the Netherlands. Tel.: +31 2436 14567; Fax: +31 2436 68754; E-mail address: [Marcel.Verbeek@radboudumc.nl](mailto:Marcel.Verbeek@radboudumc.nl)

**Index**

**Figures**

**Figure S1:** Correlation of 13 differentially expressed protein biomarker candidates with clinical and imaging biomarkers in the PEA exploration study.

**Figure S2:** Correlation of ELLA CSF NFL levels in the validation study, with clinical and imaging biomarkers.

**Figure S1: Correlation of 13 differentially expressed protein biomarker candidates with clinical and imaging biomarkers in the PEA exploration study.** Values given represent correlation coefficients of significant Spearman correlations between variables (p ≤ 0.05), and are colour-coded from 1.0 (blue) to red (-1.0). Correlations which were not significant are not shown. **** p ≤ 0.0001, *** p ≤ 0.001, ** p ≤ 0.01, * p ≤ 0.05. LP = lumbar puncture, MoCA = Montreal Cognitive Assessment, ICH = intracerebral haemorrhage, CMB = cerebral microbleed, cSS = cortical superficial siderosis, CAA SVD = cerebral amyloid angiopathy small-vessel disease related burden score.


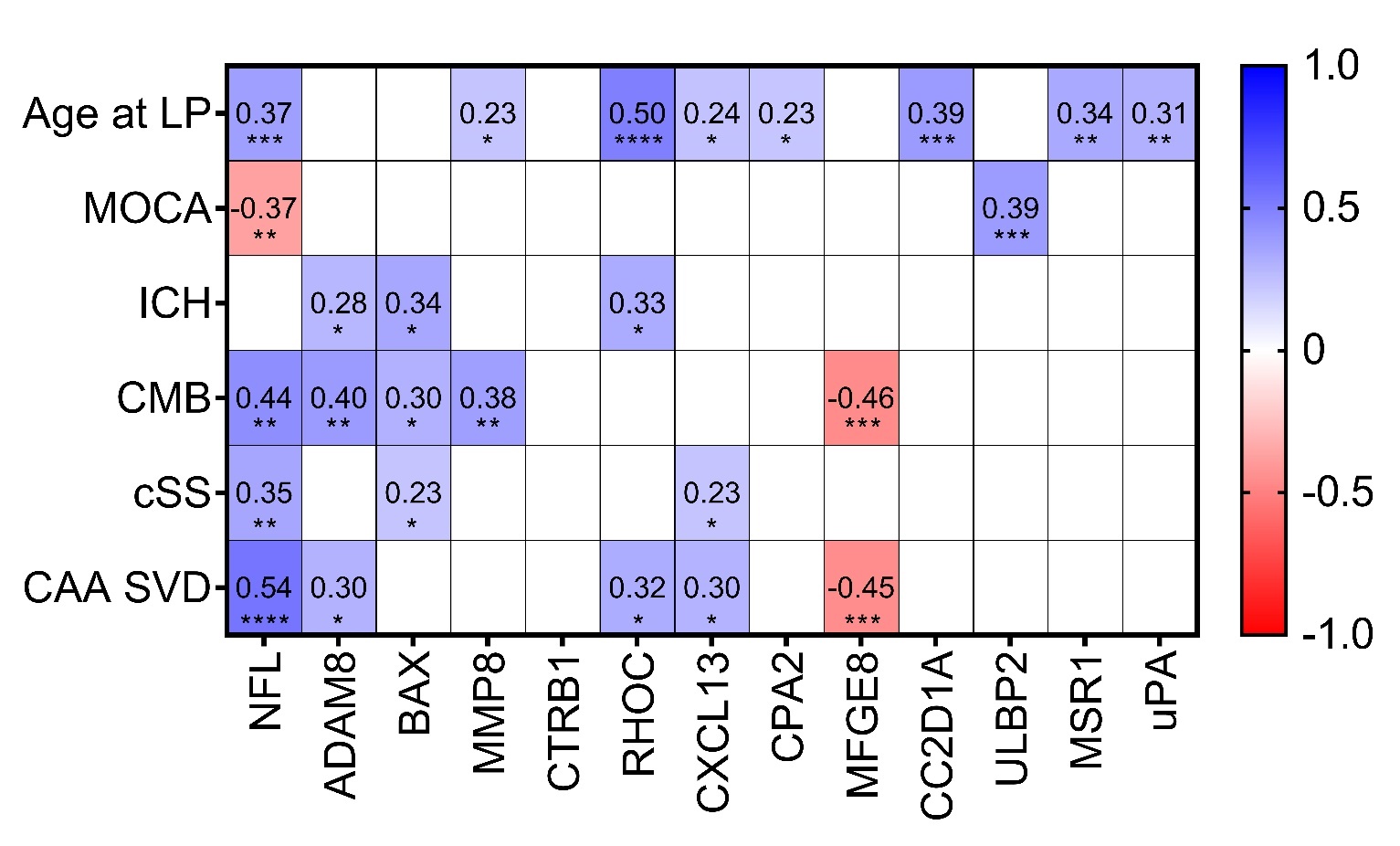


**Figure S2: Correlation of ELLA CSF NFL levels in the validation study, with clinical and imaging biomarkers.** Values given represent correlation coefficients of significant Spearman correlations between variables (p ≤ 0.05), and are colour-coded from 1.0 (blue) to red (-1.0). Correlations which were not significant are not shown. **** p ≤ 0.0001, *** p ≤ 0.001, ** p ≤ 0.01. LP = lumbar puncture, MoCA = Montreal Cognitive Assessment, ICH = intracerebral haemorrhage, CMB = cerebral microbleed, cSS = cortical superficial siderosis, CAA SVD = cerebral amyloid angiopathy small-vessel disease related burden score.


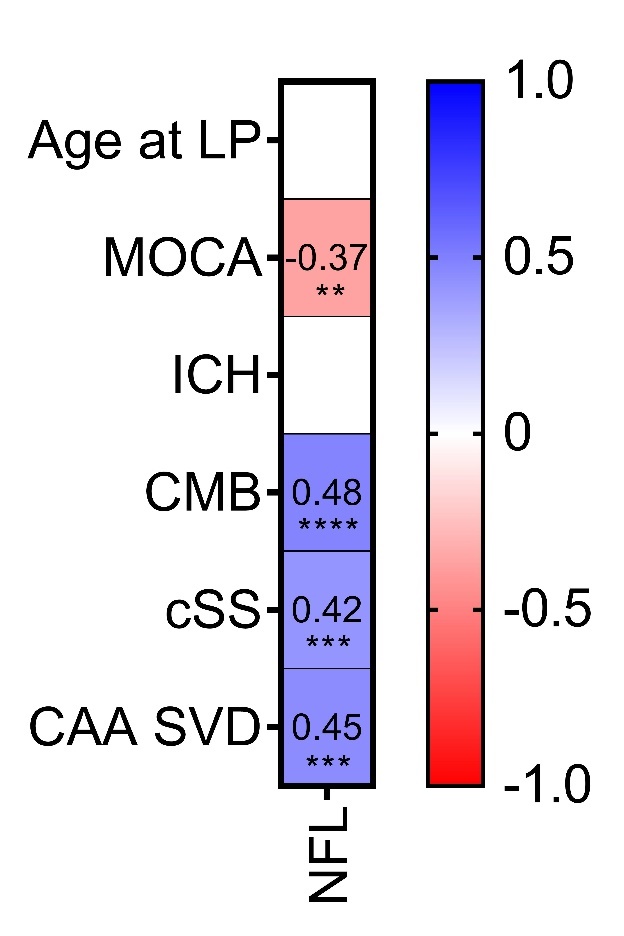

Supplement: Supplementary file 1 — Supplementary Material 1 [file 13195_2024_1473_MOESM1_ESM.docx]
